# Effect of At-Home Versus Over-the-Counter Bleaching Agents on Enamel Color, Roughness, and Color Stability

**DOI:** 10.7759/cureus.39036

**Published:** 2023-05-15

**Authors:** Rasha R Basheer, Dalia M Abouelmagd, Abeer Alnefaie, Raghad Baamer

**Affiliations:** 1 Restorative Department, Faculty of Dentistry, King Abdulaziz University, Jeddah, SAU; 2 Conservative Department, Faculty of Dentistry, October University for Modern Sciences and Arts, Giza, EGY; 3 Restorative Department, Faculty of Dentistry, Cairo University, Cairo, EGY; 4 General Dentistry Department, Faculty of Dentistry, King Abdulaziz University, Jeddah, SAU

**Keywords:** carbamide peroxide, hydrogen peroxide, at-home bleaching, over-the-counter products, color stability, surface roughness, tooth bleaching

## Abstract

Background

This study was designed to evaluate the efficacy of standard at-home bleaching agents in comparison to new over-the-counter (OTC) products on human enamel regarding color change, color stability, and surface roughness.

Methodology

A total of 80 extracted adult human maxillary central incisors were prepared and arbitrarily divided into the following four equal groups (N =20): group A: at-home opalescence boost containing 15% carbamide peroxide (CP); group B: crest whitening strips containing 6% hydrogen peroxide (HP); group C: light-emitted diode (LED) home tray with 20% CP + 4% HP; and group D: white and black toothpaste containing active charcoal components. Tooth color was measured using a spectrophotometer. Enamel surface roughness using a three-dimensional optical profilometer was measured before and after the bleaching procedure. To assess color stability, each bleached group was further subdivided into two equal subgroups (n = 10) according to the immersion media of either coffee or tea. Finally, the color was measured after 24 hours of immersion.

Results

All groups showed improvement in color from the baseline. The crest whitening strips group showed the lowest color improvement in comparison to all other groups. After staining, group C showed the lowest mean color change value ∆E2. No statistically significant difference was noted in surface roughness among all groups.

Conclusions

All OTC bleaching products as well as at-home bleaching improve teeth color and increase roughness on the enamel surface. Staining media has an adverse effect on the teeth after bleaching. The LED home tray showed a better whitening effect and color stability after bleaching.

## Introduction

Patients’ demand for cosmetic esthetic dentistry and white teeth, including teeth bleaching, has become extremely high in the last decade [1]. This has created a need in daily dental practice and led to increased awareness among dental clinicians about different bleaching procedures. A combination of intrinsic and extrinsic factors can directly influence teeth color. Intrinsic stains can result from enamel and dentin alteration, whereas extrinsic stains are directly related to deposition from colored food, drinks, or tobacco stains on the surface of teeth [2]. Various treatment options are available for discolored teeth, including vital tooth bleaching, whitening trays, teeth polishing, enamel microabrasion, non-vital tooth bleaching, veneers, and full-coverage crowns [3]. Over-the-counter (OTC) tooth whitening products have several advantages such as low price, availability, and ease of use solely at home by the patient. OTC and at-home teeth bleaching are different as OTC bleaching products involve only the patient purchasing and using them independently. The decision is entirely patient-dependent without any diagnosis of the causes of discoloration [4].

Teeth bleaching is a process of brightening teeth color and is considered a conservative treatment as it does not involve any cutting of human enamel. Its mechanism of action is an oxidation reaction utilizing hydrogen peroxide (HP) diffusion into the tooth surface to produce free radicals that modify the organic pigment molecules in the enamel surface [5]. Moreover, the exact mechanism of teeth whitening using HP is not entirely clear as it has many factors affecting the reaction rather than the active oxygen. It can be also affected by reaction conditions, such as temperature, light, and pH [6]. HP has a strong oxidizing impact and produces other highly powerful bleaching agents, including per hydroxyl anions (HO_2_^−^) and hydroxyl radicals (OH^−^) [7,8]. In turn, carbamide peroxide (CP) breaks down into HP and urea, and the latter results in the denaturalization of the proteins amelogenin and enamelin, which are present in the matrix of the substance found in between the enamel prisms. This might induce microstructural alterations and likely increase enamel permeability. On the other hand, urea promotes alkalization, which might lead to less demineralization [8,9].

Utilizing lasers or light photochemically as initiators for the bleaching reaction leads to a large increase in hydroxyl radicals from HP [10]. Thereafter, peroxide molecules’ free radicals pass through the enamel until it reaches the enamel-dentine junction and dentine. These molecules can react with colored organic materials and cause the brightening of tooth color [11]. CP is recommended for at-home bleaching due to its safety and long-term successful results [12]. However, many patients are not comfortable using a fabricated tray for a long time and are anxious about the gel applied in the tray as well [13,14]. Since the beginning of the 2000s, OTC bleaching products have been available in the market at a lower cost compared to dentist-supervised management of teeth discoloration [15]. Nowadays, different products are found in the market as gels, dentifrices, mouthwashes, paint-on product formats, light-activated home devices, and whitening strips [16].

Thus, the objective of this study was to assess the efficacy and adverse effects of OTC products on human enamel in comparison to standard dentist-supervised home bleaching regarding tooth color, enamel surface roughness, and color stability. The following null hypotheses were tested: first, there is no significant difference in terms of color change between at-home bleaching agents and three OTC tooth-whitening products. Second, immersion in coffee and tea after bleaching will not influence the effectiveness of bleaching. Finally, third, there is no significant difference in terms of surface roughness between at-home bleaching agents and three OTC tooth-whitening agents.

## Materials and methods

Preparation of specimens

**Figure 1:**
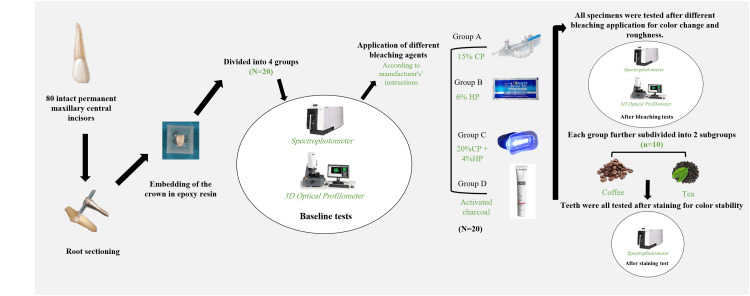
Illustration of the study design. CP = carbamide peroxide; HP = hydrogen peroxide

In this study, 80 freshly extracted human maxillary central incisors were collected and warehoused in 0.1% thymol solution and used within a maximum of four weeks after extraction. The teeth were extracted and collected under the protocol approved by the Ethical Committee of King Abdulaziz University, Jeddah, Saudi Arabia (approval number: REC 146-12-20). All teeth were cleaned and polished with prophylactic paste to remove any extrinsic stains. Subsequently, all teeth were inspected under a stereomicroscope to eliminate any crack, decay, fracture, previously restored, or structural deformities. A low-speed, double-sided diamond disc (Isomet Buehler Ltd, Chicago, IL USA) was used to perform sectioning of the roots 1 mm below the cementoenamel junction, and then the remaining coronal portions were further sectioned into labial and palatal halves. A water coolant was used in all sectioning processes. The labial halves of teeth were individually embedded inside the self-curing acrylic resin blocks measuring 20 × 20 mm. The middle third of the labial halves of teeth were barely flattened and smoothened with the labial surface exposed for application of bleaching treatment (Figure 1). Blocks were coded and arbitrarily divided into four equal groups (N = 20) according to the different bleaching agents used. Group A (control): opalescence containing 15% CP; group B: crest whitening strips containing 6% HP; group C: light-emitted diode (LED) home tray with 20% CP + 4% HP; and group D: white and black toothpaste containing active charcoal components as active whitening ingredients (Table 1). All teeth were stored during all stages of the testing in distilled water to avoid dehydration [17].

**Table 1:** Whitening products used in this study.

Group code/Materials/Manufacturers	Composition	Delivery method/Duration of use
Group A/Opalescence^TM ^PF/Ultradent Company, USA	Glycerin, water (aqua), 15% carbamide peroxide, xylitol, carbomer, PEG-300, sodium hydroxide, EDTA, flavor (aroma) mint and melon flavors only, potassium nitrate, sodium fluoride	A micro-brush was used to place and cover all the facial surfaces of each specimen for four hours per day for 10 days
Group B/Crest 3D whitening strips/Procter & Gamble Company (P&G), USA	Water, glycerin, 6.5 % hydrogen peroxide, carbomer, polyvinylpyrrolidone (PVP), PEG, acrylates copolymer, sodium hydroxide, sodium saccharin, pyrophosphate, polyethylene, polypropylene	The whitening strip was cut in the proper size to the facial surface of the tooth to ensure covering the whole surface for 30 minutes per day for 14 days
Group C/Light-activated teeth whitening/Spa Dent Company, Canada	Water (aqua), glycerin, 20% carbamide peroxide, 4% hydrogen peroxide, xylitol, erythritol, carbomer, dipotassium phosphate, Aloe barbadensis leaf juice, Mentha arvensis leaf oil, Melia azadirachta leaf extract, zinc citrate, calcium lactate, potassium citrate, disodium EDTA, polysorbate 80	A LED blue light pod was focused on the facial tooth surface after placing Xyliprox gel with a micro-brush for 10 minutes per day for one week
Group D/White and black toothpaste/CURAPROX, Oral Science Pro Company, Switzerland	Aqua, sorbitol, glycerin, hydrated silica, charcoal powder, aroma, argilla, decyl glucoside, cocamidopropyl betaine, sodium monofluorophosphate, tocopherol, mica, xanthan gum, hydroxyapatite (nano), titanium dioxide microcrystalline cellulose, maltodextrin, potassium acesulfame, sodium benzoate, potassium chloride, potassium sorbate, menthyl lactate, methyl diisopropyl propionamide, ethyl menthane carboxamide, zea mays starch, stearic acid, cetearyl alcohol, citrus limon peel oil, citric acid, lactoperoxidase, glucose oxidase, amyloglucosidase, tin oxide, sodium bisulfite, hydrogenated lecithin, limonen, CI75810, CI77289	Toothbrush/2:1 (toothpaste:water) ratio, a total of 16,200 cycles were done, at 40 mm/s speed and a travel length of 8 mm, with a circular motion and a load of 250 g

Bleaching procedures

All specimens in each group were bleached according to the manufacturer’s instructions.

Group A: A micro-brush was used to cover all labial surfaces of each specimen with opalescence boost gel, and then washed with distilled water. This procedure was repeatedly performed for four hours each day for 10 days.

Group B: Each whitening strip was cut in the proper size to cover the labial surface of each specimen. The application time of each strip was 30 minutes, followed by washing. This procedure was repeatedly performed for 14 days [15].

Group C: The light whitener pod was focused on the labial specimen surface after placing Xyliprox gel with a micro-brush for 10 minutes daily, and then washed with distilled water. This procedure was repeatedly performed once daily for seven days.

Group D: Teeth were brushed with a 2:1 (toothpaste:water) ratio, while they were locked in the brushing machine. A total of 16,200 cycles were done, at a 40 mm/s speed and a travel length of 8 mm with a circular motion, and a load of 250 g was set as brushing apparatus. After brushing, each specimen was rinsed.

All bleaching procedures were completed by the same operator. All specimens were washed and then warehoused in distilled water at a temperature of 37°C after the bleaching procedure.

Color measurement

The baseline color measurement, before any bleaching procedure, was performed for all specimens using a spectrophotometer (Color Eye 7000A, X-rite, Grand Rapids, MI, USA), which is an analytical instrument used to quantitatively measure the transmission of visible light, ultraviolet light, or infrared light by three coordinates L*, a*, and b*. L* represents the darkness/brightness with values ranging from 0 to 100; a* values are arranged from -80 (green) to +80 (red) and denotes the green-red element; and b* represents the blue-yellow elements with values ranging from -80 (blue) to +80 (yellow) [18]. Subsequently, the color difference for periods between before and after treatment (∆E1) was recorded: ∆E* = [(∆L*)2 + (∆a*)2 + (∆b*)2] 1/2, where ∆E1 = color change; ∆L = Lfinal - Linitial; ∆a = afinal - initial; and ∆b = bfinal - binitial [17].

Enamel surface roughness measurement

Using a non-contact profilometer (Bruker Contour GT-K, Tucson, AZ, USA), the enamel surface roughness of all tested specimens was recorded before and after bleaching treatment. The profilometer has a three-dimensional (3D) optical microscope with a nano-lens atomic force microscopy module, which uses a Vision 64 (Bruker, Tucson, AZ, USA) to regulate the device parameters, resulting in a high-resolution outcome of the scanned specimen surface.

Staining process

According to the staining solution, either coffee or tea, each group was arbitrarily subdivided into two equal subgroups (n = 10). Immediately after bleaching, specimens were soaked in a vial containing the solution and kept in the incubator at 50°C in the coffee and tea groups for 24 hours which simulated one-month consumption. For the coffee group, 3.6 g of the coffee powder was liquefied in 500 mL of boiled distilled water (Nescafe Red Mug, Nestle Brazil, Araras, Brazil). For the tea group, 2 g of two bags of prefabricated black tea was submerged in 500 mL of boiled purified water for 10 minutes (Ahmed Tea, English tea, London, UK). The specimens were rinsed and dried after storage. Then, the spectrophotometer was used to measure the color after staining ∆E2 [18,19].

Statistical analysis

Means and standard deviations were calculated for each individual group for all tests. Data were then checked for normality via the Kolmogorov-Smirnov and Shapiro-Wilk tests, with the data showing a parametric (normal) distribution. To collate more than two groups in non-related samples, a one-way analysis of variance followed by the Tukey post hoc test was used. To collate between two groups in related samples, the paired-sample t-test was used. The significance level was set at p-values ≤0.05. Statistical analysis was performed using SPSS Statistics Version 20 for Windows (IBM Corp., Armonk, NY, USA).

## Results

Color changes

**Figure 2:**
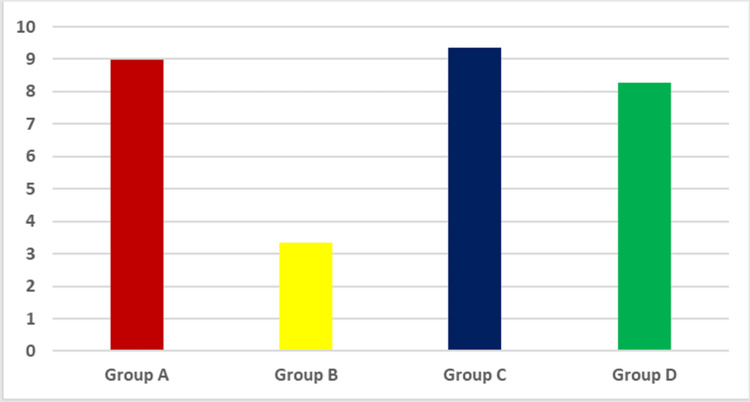
Bar chart representing mean values of ΔE1 after bleaching for different groups

**Figure 3:**
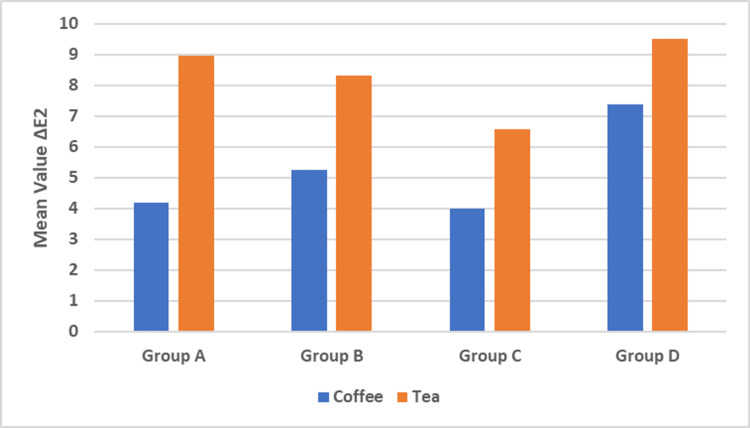
Bar chart representing ∆E2 comparison between each group in tea and coffee staining mediums.

The mean and standard deviation values were calculated for each group in each test. Table (2) and figure (2) display mean and standard deviation values of color change before and after bleaching while table (3) and figure (3) display mean and standard deviation values of color change after bleaching and immersion in staining solutions.

After application of different bleaching agents (ΔE1)

There was no statistically significant difference in mean color chage values between group A and each of group C and group D at p<0.001, meanwhile there was as tatistically significant difference between group C and D at p<0.001.. A statistically significant difference was found between group B and each of group A, group C and group D at p<0.001. , where group B showed the lowest mean color change value of 3.34 and group C showed the highest mean color change value of 9.35.

**Table 2:** The mean, standard deviation (SD) values of ∆E1 of different groups. Means with different small letters in the same column indicate significant difference, *; significant (p<0.05)

Variables	∆E1	
	Mean	SD
Group A	8.97 ab	0.97
Group B	3.34 c	0.64
Group C	9.35 a	0.95
Group D	8.27 b	0.92
p-value	<0.001*	

Immersion in different staining solutions after bleaching (ΔE2)

There was a statistically significant difference between all tested coffee and tea groups. All groups immersed in coffee showed a statistically significant lower mean color change values compared to all groups immersed in the tea, with a p-value <0.001 (Table 3 and Figure 3).

**Table 3:** The mean and standard deviation (SD) values of ∆E2 of different groups. Means with different small letters in the same column indicate significant differences and means with different capital letters in the same row indicate significant differences. * = significant (p < 0.05).

Variables	∆E2				
	Coffee		Tea		P-value
	Mean	SD	Mean	SD	
Group A	4.18^ acB^	0.51	8.97^ abA^	0.72	<0.001*
Group B	5.25^ bB^	0.98	8.33^ bA^	0.69	<0.001*
Group C	4.01^ cB^	0.64	6.58^ cA^	0.71	0.002*
Group D	7.38^ aB^	0.26	9.50^ aA^	0.68	0.001*
P-value	<0.001*		<0.001*		

Effect of Coffee on Color Stability in Different Groups After Bleaching (∆E2)

Regarding mean color change values after bleaching, a statistically significant difference was found between group A and group B, while no statistically significant difference was found between group A and groups C and D. Moreover, a statistically significant difference was found between group B and both groups C and D (p < 0.001). Meanwhile, a statistically significant difference was found between group C and group D, where group D showed the highest mean color change value of 7.38, and group C showed the lowest mean color change value of 4.01.

Effect of Tea on Color Stability in Different Groups After Bleaching (∆E2)

Regarding mean color change values, there was no statistically significant difference between group A and group B and between group A and group D. Meanwhile, a statistically significant difference was noted between group B and group D (p < 0.001). On the other hand, there was a statistically significant difference between group C and groups A, B, and D (p < 0.001), where group C showed the lowest mean staining value of 6.58, and group D showed the highest mean staining value of 9.5.

Enamel surface roughness

**Figure 4:**
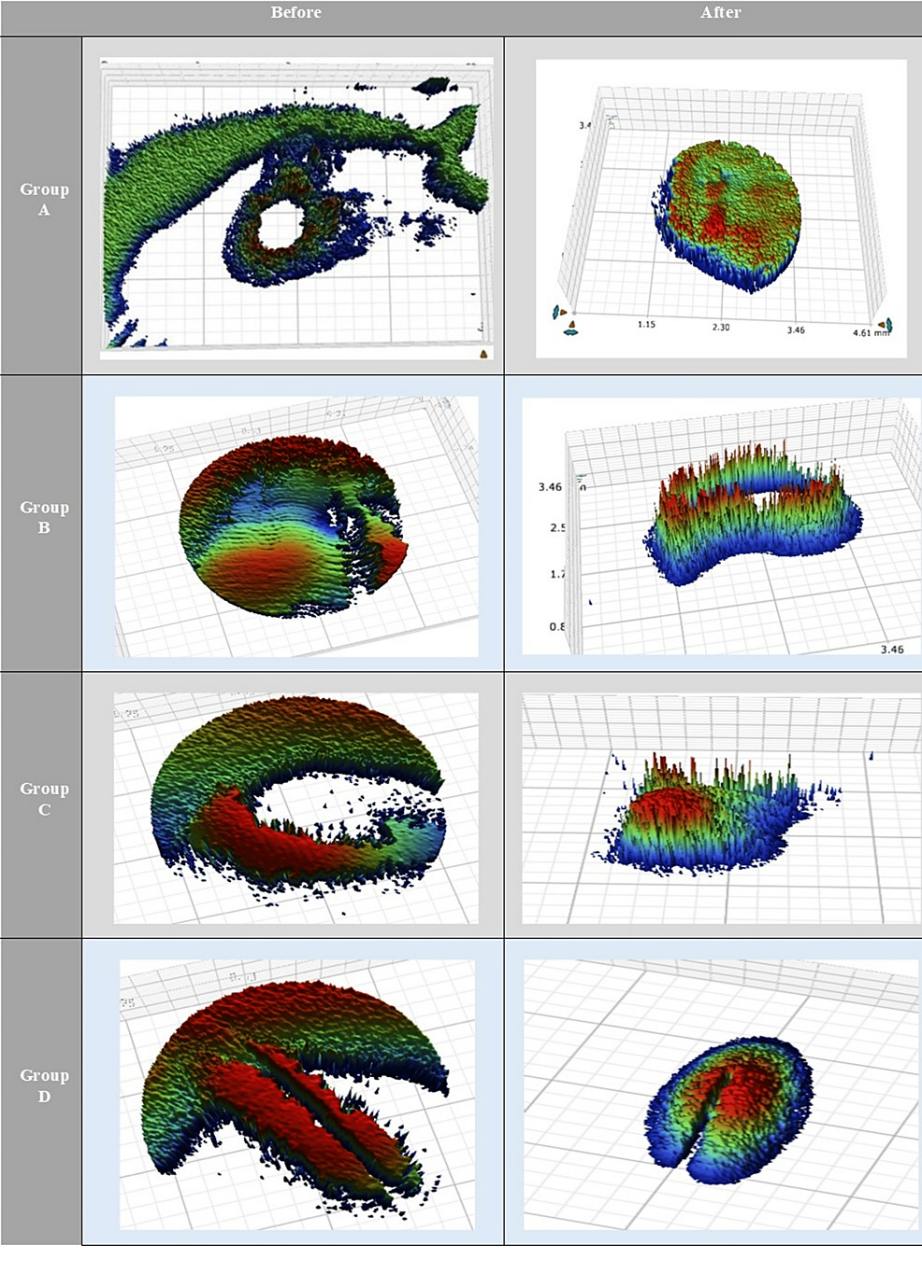
Surface roughness before and after different bleaching treatments using a three-dimensional optical profilometer. Three-dimensional optical profilometer images before and after the application of bleaching gels. Group A: opalescence boost containing 15% carbamide peroxide, group B: crest whitening strips containing 6% hydrogen peroxide, group C: a light-emitted diode home tray with 20% carbamide peroxide + 4% hydrogen peroxide, and group D: white and black toothpaste containing active charcoal components.

**Figure 5:**
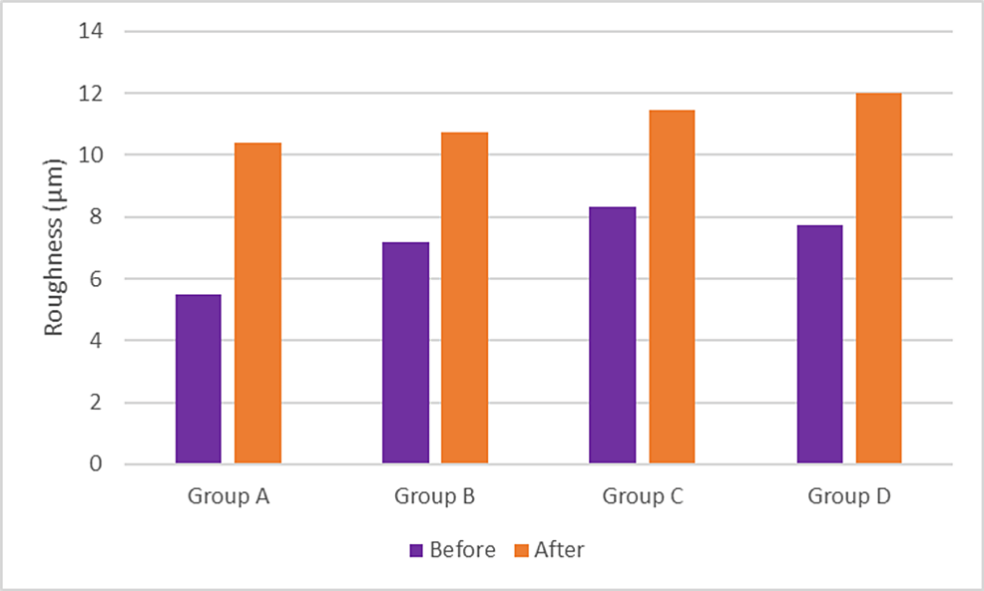
Bar chart representing roughness for different groups before and after bleaching.

Regarding the mean surface roughness values, a statistically significant difference was noted between group A and each of groups B, C, and D before bleaching (p = 0.006). Meanwhile, no statistically significant difference was noted between all tested groups after bleaching (p = 0.07). All tested groups showed a statistically significant increase in mean surface roughness values after bleaching (p < 0.001) (Table 4 and Figures 4,5)

**Table 4:** The mean, standard deviation (SD) values of Roughness of different groups Means with different small letters in the same column indicate significant differences and means with different capital letters in the same row indicate significant differences. * = significant (p < 0.05); ns = non-significant (p > 0.05).

Variables	Roughness				
	Before		After		P-value
	Mean	SD	Mean	SD	
Group A	5.51^ bB^	2.20	10.41^ aA^	0.29	<0.001*
Group B	7.18^ abB^	2.47	10.75^ aA^	2.65	<0.001*
Group C	8.31^ aB^	2.17	11.44^ aA^	1.93	<0.001*
Group D	7.75^ aB^	3.21	12.02^ aA^	2.46	<0.001*
P-value	0.006*		0.070ns		

## Discussion

At-home teeth bleaching is a dentist-supervised procedure that can lighten the color of the teeth. This esthetic treatment can affect the patient’s teeth color and accordingly enhance their self-esteem [20]. On the other hand, the main attraction of OTC whitening products is that they can be easily purchased and applied independently by patients without the need for a dentist’s supervision [21]. In this study, the color difference after bleaching and staining was assessed using a spectrophotometer. It is considered to be an objective approach that provides more dependable and precise outcomes [22] than the visual perception of color and helps eliminate personal factors that may interfere with the results [23]. After the application of each bleaching product, specimens were kept in distilled water, thus simulating the oral cavity condition [24,25]. Coffee and tea were selected as immersion solutions to test the color stability of the teeth after the bleaching process as they are known to be the most frequently consumed beverages by a large sector of the population and have a significant potential for teeth staining [26].

Several techniques can be used to assess the effectiveness of teeth whitening processes. In this regard, the spectrophotometric and profilometric methods employed in our study are unbiased, precise, and repeatable [27,28]. In this investigation, we followed the whitening instructions provided by the manufacturers, keeping the samples immersed in distilled water at 37 °C between treatments. Distilled water was utilized to simulate the flushing action of salivary flow as artificial saliva is not considered a therapeutically relevant environment [29]. To avoid subjectivity, color was assessed using the CIE Lab color system, which classifies colors based on human observation. Color difference value (ΔE) is deemed clinically detectable in this method when it equals or exceeds 3.3 [30].

At-home and OTC bleaching agents exhibited a statistically significant increase in the whitening effect. This can be interpreted by the fact that the enamel histological structure is weaker in the presence of acidic substances such as peroxide bleaching chemicals [31]. Because bleaching treatments enhance enamel microporosities [32], acid permeates enamel deeper and oxidizes more stain-containing molecules as a result causing an improved whitening effect. There was a statistically significant difference between the means of color changes ΔE1 after the application of the different bleaching treatments, OTC and at home, so the first null hypothesis was rejected. The crest whitening strips group (group B) showed the least color change (ΔE1 = 3.34), which might be attributed to the maladaptation of the strip on the tooth [33]. In addition, it could be correlated to the low HP concentration percentage in the whitening strip leading to less whitening effect in comparison to the other products of higher percentages [8]. This supports earlier research that reported that longer enamel bleaching times produced superior color improvements independent of the bleaching chemical concentration [34-36].

Drinking stain-producing beverages may reduce the effectiveness of bleaching, and the likelihood of discoloration appears to be mostly dependent on the staining agent [37]. The results of this study showed statistically significant enamel discoloration of bleached teeth after immediate immersion in coffee and tea, so the second null hypothesis was rejected. These results are in line with some studies which revealed increased vulnerability to staining right after bleaching [38-41]. A rough enamel surface due to the imperfections resulting from the bleaching process is more susceptible to stains [39], and, hence, the food colors could stick more significantly to those rough surfaces [40]. Due to their high pigment content, coffee and tea have the highest potential to stain teeth [41-43]. The low pH levels of coffee and tea may cause enamel disintegration and increase surface porosity, which can lead to tooth discoloration [37,44]. Moreover, anionic polyphenols, which are present in large quantities in tea and coffee, may interact with cationic salivary pellicles to generate thicker layers of stained materials, resulting in tooth discoloration [45,46]. These findings did not coincide with other studies suggesting that it is unnecessary to avoid drinking coffee following in-office bleaching [47-49]. Disparities in the research design, the measuring period, the application procedures, and the bleaching agents are only a few examples of the possible causes of discrepancies [50]. The tea groups showed statistically significantly higher discoloration to bleached teeth than coffee. This result was in agreement with studies concluding that tea causes more staining than coffee [51] due to the presence of tannins, which permeate the inherently porous enamel and produce an undesirable brown tint. In addition, the process of bleaching is considered to occur through the enamel, enabling tiny molecular-weight particles to flow via its structure, which might explain why pigments found in substances such as coffee with high molecular weight might not be able to permeate the enamel surface [21,25].

On the other hand, the LED home tray group (group C) showed the most minor discoloration after immersion in coffee and tea, which may be attributed to the greater bleaching effectiveness induced by LED lighting. Accordingly, there could be less influence by immersion in different staining solutions [52].

Moreover, the toothpaste group (group D) showed a high statistically significant whitening effect and the highest statistically significant staining potential after bleaching. This is in accordance with the conclusion that activated charcoal-based toothpaste generates the most abrasive potential on the enamel surface, thus leading to an increase in roughness which accelerates the deposition of stains. According to reports, peroxide-based bleaching chemicals cause morphological changes in enamel due to altered inorganic and organic composition, increasing its surface roughness [53-56]. Moreover, while free radicals produced during bleaching react non-selectively with the organic structure of dental tissues, they can enhance porosity on tooth surfaces [53,57,58].

All bleaching agents tested in this study showed a significant increase in the enamel roughness after bleaching, with no significant difference between tested groups for mean roughness data (p = 0.07); hence, the third null hypothesis was accepted. This result was in agreement with previous studies [53,56-59], which concluded that utilizing bleaching gels with various peroxide concentrations would lead to changes in the enamel surface that would influence its roughness. The results were in disagreement with other studies [43,60-62], which indicated that the bleaching procedure may not increase the enamel micro-roughness. Many factors could be attributed to the variation in results stated in the literature such as different lighting sources used, tooth types chosen for the study, the use of materials in the market, and specimen storage media. In our investigation, the samples were kept in distilled water, which prevents saliva from having a remineralizing impact. The pH of the product used is another factor that can help explain the discrepancies in the published results. In this aspect, some authors believe that the product’s pH plays a greater role in regulating morphology and roughness changes than HP content. [62-65]. In the current study, all active ingredients used in bleaching products were different. Group A consisted of 15% CP, crest 3D whitening strips contained 6.5% HP, light-activated teeth whitening contained 20% HP and 4% CP, and white and black toothpaste contained hydrated silica and charcoal powder. The different activators, their concentration, and pH, besides the amount of time the bleaching agent was in contact with the surface of the enamel, may have led to the increase in enamel roughness [55-57]. Nonetheless, it has recently become clear that polishing, fluoridation, and remineralizing treatments can greatly reduce the roughness of bleached enamel [57].

Study limitations

It is hard to simulate the complex oral environment. The in-vitro design of this study is considered to be a limitation of this study. in addition, color stability depends on the consumption of staining beverages and food, which could vary according to personal preference and is hard to be unified. Further in-vivo studies are needed to evaluate the color stability and effect of the OTC bleaching products available in the market on human enamel.

Clinical implications

Taking into consideration how the bleaching agents affect the surface of the enamel, the LED home tray has shown promising results in teeth bleaching, time efficiency, and color stability, hence, further investigations are required. Clinicians should encourage patients to limit drinking coffee and tea immediately following the whitening process to maintain the effectiveness of the products used.

## Conclusions

All used OTC bleaching products as well as at-home bleaching can lighten the color of the teeth. Moreover, both OTC and standard at-home bleaching products can leave the enamel surface slightly rough. Furthermore, staining media have an adverse effect on teeth after bleaching. Tea as a staining media can cause more staining than coffee. Furthermore, LED home tray could be a promising method for teeth whitening and color stability.
